# Osteomyelitis of the Patella With Extension Into Parapatellar Soft Tissues in a Six-Year-Old Boy: A Case Report

**DOI:** 10.7759/cureus.73261

**Published:** 2024-11-07

**Authors:** Abdulla Abdelwahab, Faatimah Irfaanah Muzammil, Abdulla Nidal, Mason Alnouri, Sattar Alshryda

**Affiliations:** 1 Orthopaedics and Trauma Surgery, Mohammed Bin Rashid University of Medicine and Health Sciences, Dubai, ARE; 2 General Paediatrics, Al Jalila Children's Hospital, Dubai, ARE; 3 Paediatric Orthopaedics and Trauma Surgery, Al Jalila Children's Hospital, Dubai, ARE

**Keywords:** antibiotics, case report, infection, magnetic resonance imaging (mri), osteomyelitis, patella, pediatric, staphylococcus aureus

## Abstract

Acute osteomyelitis (OM) of the patella is an exceptionally rare condition in children. The rarity of this condition, coupled with its nonspecific symptoms and varied clinical presentations, makes early diagnosis challenging and often results in delayed treatment. Prompt identification and initiation of antibiotic therapy are essential for a full recovery and to prevent the disease from progressing to a chronic, more severe form. This case report discusses a previously healthy six-year-old boy who developed OM of the left patella following a fall from standing height after tripping three days prior to his presentation at the emergency department. Due to the unusual and infrequent nature of patellar OM, it is often overlooked as a potential cause of knee pain in pediatric patients. Clinicians must consider patellar OM in the differential diagnosis for children presenting with knee pain. Accurate and timely diagnosis relies on thorough clinical assessments, laboratory testing, and appropriate imagining techniques (i.e., X-ray and magnetic resonance imaging (MRI)). Early identification and appropriate treatment are critical in preventing the infection from spreading within the joint and ensuring a favorable outcome.

## Introduction

Osteomyelitis (OM) is one of the most common musculoskeletal infections in children, primarily caused by *Staphylococcus aureus*. This bone infection can occur through hematogenous spread or direct bacterial inoculation following surgery or open fractures [1]. Hematogenous OM typically affects the long tubular bones in children, with less frequent involvement of cuboidal, vertebral, and pelvic bones [2]. However, acute OM of the patella is exceptionally rare, accounting for only 1.1% of cases reported in the literature [3]. The rarity of patellar OM, combined with its nonspecific symptoms and varied clinical presentations, poses significant challenges for early diagnosis and treatment.

Timely identification and initiation of antibiotic therapy are critical to achieving full recovery and preventing the disease from advancing to a chronic and more severe state [4]. This report presents the case of a previously healthy six-year-old boy who developed OM of the left patella following a fall from a standing height incurring minimal bruising. The incident occurred three days before his presentation at the emergency department. We detail his clinical presentation, diagnostic process, treatment, and recovery, highlighting the importance of considering patellar OM in the differential diagnosis of knee pain in children.

## Case presentation

A previously healthy six-year-old boy presented to the emergency department with left knee pain that began three days ago following a fall from standing height.

One day after the incident, the child's mother noticed her child experiencing pain and observed noticeable swelling with no bruising in the left knee, which worsened progressively, leading to a reduced range of motion (ROM) and an inability to stand or bear weight on the affected limb. On presentation, the child's temperature was 38°C and had been consistently elevated for the past two days, as reported by the mother. Additionally, the child experienced abdominal pain, loose stools, and fever as reported by the mother. On examination, the left knee was tender to touch, with evident erythema and warmth. The patient only had 0-90 degrees of ROM with painful movement.

Laboratory evaluation revealed a normal white blood cell (WBC) count but high inflammatory markers as shown in Table 1. 

**Table 1:** Values of the inflammatory markers taken on the day of admission

Inflammatory marker (reference range and units)		Value on the day of admission
White blood cells (5-15×10^3^/uL)	13.6	
C-reactive protein (0-5 mg/L)	254.8	
Procalcitonin (<0.5 ng/mL)	2.17	
Erythrocyte sedimentation rate (0-20 mm/1 hr)	59	

**Figure 1:**
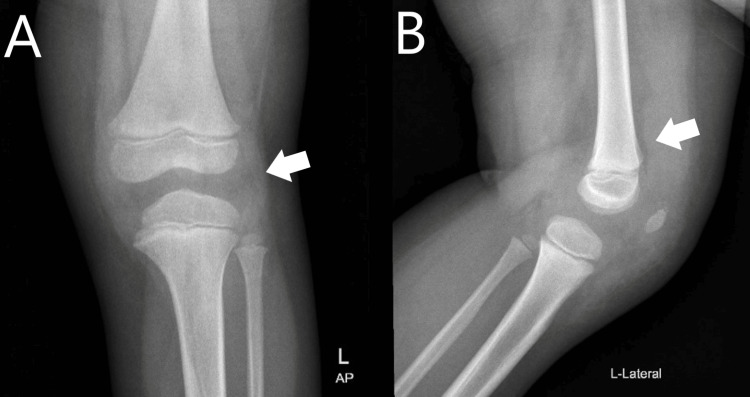
Anteroposterior (A) and lateral (B) views of the left knee with the arrows showing periarticular soft tissue swelling (arrow in A) and distension of the suprapatellar fat pad (arrow in B) suggesting joint effusion AP: anteroposterior; L: left

**Figure 2:**
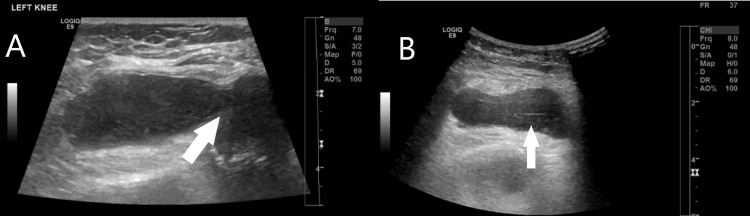
Ultrasound of the left knee showing different cuts (A and B) where the arrows point at periarticular soft tissue swelling. Distension of the suprapatellar bursa (arrow in B) is evident. Synovial thickening with minimal fluid in the knee joint (arrow in A) is also noted

X-ray of the left knee (Figure 1) was done and it reported periarticular soft tissue swelling and distension of the suprapatellar fat pad pointing towards joint effusion. Periarticular soft tissue swelling with distension of the suprapatellar bursa and synovial thickening with minimal fluid in the knee joint were also noted on ultrasound (US) (Figure 2).

On the first day after admission, a multidisciplinary discussion took place involving general pediatrics, orthopedics, and radiology. It was agreed to aspirate the knee yielding approximately 30 cc of yellow and turbid joint fluid, which was then sent for cell counts and culture analysis. Meanwhile, a repeat blood test resulted in a C-reactive protein level of 268.1 mg/L. Given the unchanged clinical picture, persistent fever, increasing inflammatory markers, and radiological findings of infection, the child was scheduled to undergo an arthroscopic washout and debridement of his left knee the following day (the second day of admission).

During the surgical procedure, employing aseptic measures, a trocar was introduced into the soft area on the lateral aspect of the knee. Approximately 15 cc of yellowish fluid mixed with blood was visibly drained. Extensive irrigation with 3 liters of saline was performed, and samples for fluid microscopy and culture were collected, along with a specimen for histopathology. Additionally, synovial fluid was obtained for cell counts and comprehensive analysis (i.e., culture, crystals, etc.).

Following the recommendation from the general pediatric and infectious diseases teams and judging by the patient's presentation, he was placed on intravenous (IV) vancomycin empirically, and the plan of care involved continuing the IV antibiotics for two weeks. The patient was closely monitored with daily observations, and laboratory tests were scheduled to be repeated biweekly. Additionally, the patient received daily visits from the physiotherapist to attain full ROM in his left knee, aiming to prevent any stiffness in the joint.

The fluid culture analysis was positive for a heavy methicillin-susceptible *Staphylococcus aureus* (MSSA) growth. IV vancomycin was switched to IV flucloxacillin, but it caused a burning sensation on administration, so it was changed to IV cefazolin. The patient was on IV antibiotics for a total of two weeks.

Laboratory tests were carried out multiple times over different intervals postoperatively, revealing a considerable drop in all inflammatory markers by discharge day as shown in Table 2.

**Table 2:** The trend of inflammatory markers from admission to discharge suggesting resolution of the infection *: Reference range and units are in brackets

Days	C-reactive protein (0-5 mg/L)*	Procalcitonin (<0.5 ng/mL)*	Erythrocyte sedimentation rate (0-20 mm/1 hr)*
Day 0 (admission day)	254.8	2.17	59
Day 1	268.1	1.53	68
Day 2	Surgical intervention		
Day 3	164.7	0.94	65
Day 4	98.1	0.36	64
Day 9	78.1	0.05	2
Day 10 (discharge day)	25.9	0.05	2

**Figure 3:**
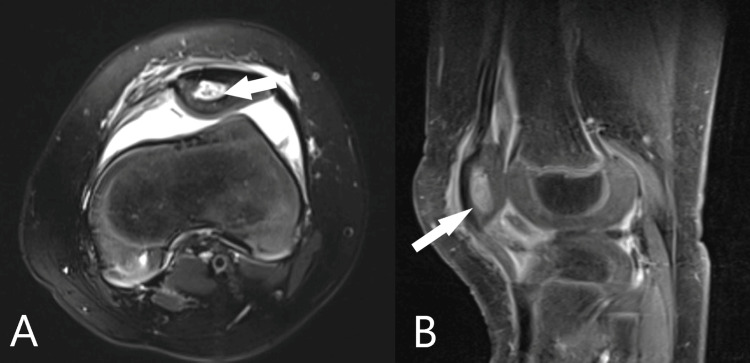
Magnetic resonance imaging with axial (A) and sagittal (B) cuts of the left knee showing patellar osteomyelitis with associated cortical destruction (arrow in B) and a small communicating sinus tract (arrow in A)

A few days after the surgery, there were prepatellar swelling, rash, and tenderness; therefore, magnetic resonance imaging (MRI) of the left knee was conducted to rule out OM around the knee. The study confirmed patellar OM with a hairline-like sinus extending to the knee's medial parapatellar soft tissues (Figure 3). However, the amount of pus was very trivial, so the IV antibiotic treatment was continued.

Given the improved clinical condition of the patient and the declining trend of his inflammatory markers, he was released from the hospital a week after the surgery with a prescription for oral flucloxacillin, which continued for a total duration of four weeks (making it a six-week course of antibiotics). Subsequently, he received ongoing follow-up care in the outpatient clinic until his discharge from the pediatric orthopedics department after one year. A phone call with his mother a year and a half later confirmed the return of his knee to a normal ROM including the return to sports.

## Discussion

Septic arthritis of the knee is relatively common in pediatric orthopedic practice, and its association with OM of the distal femur or proximal tibia is well-documented in the literature. However, to the best of our knowledge, this is the first case report that links OM of the patella with septic arthritis of the knee. Accurately diagnosing patellar OM can be challenging, often resulting in delayed diagnosis due to its rare occurrence and varied clinical presentations. The initial differential diagnoses typically considered include cellulitis, bursitis, septic arthritis, and synovitis [2].

Children presenting with patellar OM may experience a range of symptoms, including subjective pain, restricted joint movement, and altered gait patterns [2]. The patella receives blood from two primary nutrient arteries, one entering the anterior surface's middle third and another at the lower pole behind the patellar ligament. A complex network of vessels from the superior and inferior genicular arteries provides a rich blood supply, and the absence of an epiphyseal growth plate in the patella seemingly reduces its susceptibility to OM [3,4]. In younger children, the thick layer of cartilage behind the ossifying section offers resistance to infection. However, as children age, the ratio of cartilage to bone decreases, potentially leading to more complicated infections such as purulent arthritis. If suppuration occurs, the pus typically surfaces anteriorly, resembling prepatellar bursitis [5].

The diagnostic process is complicated by the nonspecific nature of physical examination findings, imaging, and laboratory tests for acute OM of the patella [4]. Symptoms can range from mild to severe pain around the patella, leading to limping or restricted movement, with varying degrees of prepatellar swelling, and local inflammatory signs are not always evident. Laboratory tests may indicate a high WBC count and elevated C-reactive protein levels. Early-stage plain radiographs might only reveal soft tissue swelling in the prepatellar area, with bone destruction and sclerosis appearing later [5]. Clinicians should consider patellar OM when signs of cellulitis or bursitis in the anterior knee persist despite appropriate antibiotic treatment [3]. Diagnostic imaging techniques include radiography, radionuclide bone scans, computed tomography (CT), and MRI, with MRI being particularly valuable in confirming the diagnosis [4,6]. Aspirating the patella or prepatellar bursa for Gram stain and culture is the most effective method for identifying the causative organism, with *Staphylococcus aureus* being the most common pathogen [5]. In the case presented, the culture of the aspirate revealed a heavy growth of methicillin-sensitive *Staphylococcus aureus* (MSSA), although other pathogens have also been reported [5,7,8].

Antibiotic therapy is the cornerstone of treatment for uncomplicated cases of patellar OM, although the duration and preferred route of administration, oral or IV, vary across reports [5-10]. Guidelines for treating patellar OM are not well-defined, but most pediatric cases of hematogenous OM are managed with antibiotics. Surgical intervention, including debridement and curettage, may be necessary to remove necrotic material or sequestrum in the presence of an abscess or bone sequestration [2,5]. The decision to proceed with surgery, as well as the choice between conservative and surgical treatment, remains a subject of debate. Indications for surgery include the presence of subperiosteal and soft tissue abscesses, lack of response to IV antibiotics within 48-72 hours, and evidence of bone sequestration [4,11]. Removing all necrotic material and pus is essential for a favorable clinical outcome and to prevent the disease from recurring or becoming chronic. If pyogenic arthritis is suspected alongside OM of the patella, knee joint aspiration should precede any joint space surgery. This is important because what may appear to be purulent fluid could actually be a sterile joint effusion reactive to the OM, thus avoiding unnecessary arthrotomy that could introduce infection into the joint space [4].

## Conclusions

The rarity of patellar OM often leads to it being overlooked as a cause of knee pain in children, resulting in delayed diagnosis and increased risk of chronic progression or complications. Clinicians should consider patellar OM in pediatric patients with knee pain to ensure timely diagnosis. Accurate diagnosis relies on thorough clinical assessments, inflammatory marker testing, and imaging such as plain radiography and MRI. Early identification and appropriate treatment, ranging from antibiotics to possible surgical intervention, are crucial to prevent the infection from spreading and to avoid long-term consequences.
